# Relationship between oral health literacy and oral health-related quality of life in patients with bladder cancer

**DOI:** 10.3389/fpubh.2024.1385443

**Published:** 2024-05-23

**Authors:** Fatemeh Babadi, Ahmad Ahmadi, Mohsen Sarkarian, Maria Cheraghi

**Affiliations:** ^1^Department of Oral and Maxillofacial Medicine, School of Dentistry, Ahvaz Jundishapur University of Medical Sciences, Ahvaz, Iran; ^2^School of Dentistry, Ahvaz Jundishapur University of Medical Sciences, Ahvaz, Iran; ^3^Department of Urology, School of Medicine, Golestan Hospital, Ahvaz Jundishapur University of Medical Sciences, Ahvaz, Iran; ^4^Cancer Research Center, Department of Public Health, School of Health, Ahvaz Jundishapur University of Medical Sciences, Ahvaz, Iran

**Keywords:** quality of life, bladder cancer, DMFT, Oral, Health Literacy

## Abstract

**Introduction:**

Bladder cancer is one of the most important diseases that threatens oral and dental health due to its nature and side effects of chemotherapy. Therefore, the present study was conducted to investigate the relationship between oral health literacy and oral health-related quality of life in patients with bladder cancer.

**Methods:**

This cross-sectional study was conducted on patients with bladder cancer in Ahvaz, 2023. Subjects were selected randomly from the patients those were registered in Cancer Registry Center in Ahvaz Jundishapur University of Medical sciences and invited to Golestan Hospital for data collection through clinical evaluation, the Oral Health Literacy Adult Questionnaire (OHL-AQ), and the Oral Health Impact Profile-14 (OHIP-14PER) questionnaire. The data were analyzed using Pearson correlation coefficient, independent *t*-test, and analysis of variance.

**Results:**

The number of participants was 194. The mean oral health literacy in patients with bladder cancer was 9.74 ± 2.39, indicating insufficient oral health literacy. A significant association was observed between OHL-AQ and DMFT index, but no significant association was found between OHIP-14PER and DMFT index. Furthermore, a significant correlation was found between OHL-AQ and OHIP-14PER (*r* = −0.68) in patients with bladder cancer.

**Conclusion:**

Based on the findings of the present study, all dimensions of oral health literacy have correlation with the oral health-related quality of life in patients with bladder cancer. Therefore, adopting oral health behaviors and increasing oral health literacy can be the best way to improve the oral health-related quality of life to among patients with bladder cancer.

## Introduction

Bladder cancer is the sixth most common cancer and the ninth leading cause of cancer-related deaths worldwide. It is the most common cancer in men in the Eastern Mediterranean region and the second most common cancer among Iranian men. The sixth most common cancer in Iran is bladder cancer, with age-standard incidence rate of 8.4 per 100,000 population (1). According to a study conducted in Iran in 2020, the incidence rate of bladder cancer was approximately 1.6–115, the cumulative survival rate was 95.0%, the mortality rate was 0.84–0.5, and its prevalence in the Iranian population ranged from 4.10 to 12.8% (2). Another study examining the 12-year trend of age-standard incidence rate in Iran from 2003 to 2015 showed an increase in the incidence rate from 8.35 in 2003 to 13.57 in 2015 among men. There was also a slight increase in the age-standardized incidence rate of bladder cancer among women (2.12 in 2003 compared to 2.86 in 2015) (3).

In a study conducted by Lund Jaheim et al. in 2022, it was demonstrated that low levels of antibodies against oral bacteria *Tannerella forsythia* and *Treponema denticula* can be predictive of bladder cancer (4). In the Health Professionals Follow-Up Study conducted in 2020, Hannah Oh et al. performed a prospective analysis on a cohort of 45,185 men. Among these men, there were 563 cases of invasive bladder cancer. The study focused on individuals with a history of periodontal disease only. The findings revealed that this group had a higher risk of developing invasive bladder cancer (hazard ratio: 1.19 and a 95% confidence interval ranging from 0.98 to 1.46) (5). Furthermore, a 26-year follow-up investigation on the correlation between periodontal disease and cancer was conducted on a cohort of 19,933 male participants who were non-smokers. This study was carried out as part of the Health Professionals Follow-up Study. The results of this study show that there was a 33% increase in the risk of smoking-related cancers (lung, bladder, oropharyngeal, esophageal, kidney, stomach, and liver cancers; HR = 1.33, 95% CI: 1.07–1.65). The association was particularly strong for the risk of bladder, esophageal, and head and neck cancers, but was also increased in other smoking-related cancers (6).

On the other hand, oral-dental side effects of unconventional chemotherapy are not uncommon. For instance, nearly 40% of cancer patients undergoing chemotherapy experience undesirable reactions in the oral cavity, with approximately half of them suffering from severe mucositis that requires modification, delay, or discontinuation of treatment (4, 7). Pain caused by oral mucositis can make eating difficult for patients and potentially lead to malnutrition (7). Moreover, cancer patients often experience a decline in their oral somatosensory perception, specifically affecting their sense of taste and smell. This decline has been linked to weight loss, decreased food consumption, and a decrease in overall quality of life (8). Additionally, problems with eating and poor oral health can result in increased bacterial colonization in the mouth and an increased risk of developing pneumonia (9).

Bladder cancer is not only a threat to oral health due to the link between oral health and the risk of bladder cancer, but also because of the impact of chemotherapy and radiotherapy on the oral and dental system, it is among the most important diseases that threaten dental and oral health. Oral health literacy is one of the strategies for oral health promotion, which was identified as one of the five key measures for improving oral health at the 7th International Conference on Health Promotion by the World Health Organization (10).

The World Health Organization defines health literacy as “The cognitive and social skills which determine the motivation and ability of individuals to gain access to understand and use information in ways which promote and maintain good health” (11). Studies indicate that individuals with low oral health literacy do not take advantage of preventive, therapeutic, or informational services provided by health organizations to the community (12). Furthermore, numerous studies have shown that oral health literacy is associated with the oral health-related quality of life (13). The quality of life related to oral health is a multidimensional concept that reflects individuals’ comfort during eating, sleeping, social interactions, as well as their confidence and satisfaction with their oral health, which is influenced by functional, psychological, social, and pain or discomfort-related factors (14).

According to the increasing prevalence of bladder cancer and oral diseases resulting from chemotherapy and radiotherapy treatments, as well as the significant role of oral health literacy in the prevention and treatment of oral diseases and its impact on the oral health – related quality of life, we have conducted a cross-sectional study to investigate the relationship between oral health literacy and oral health-related quality of life, as well as the DMFT index, in patients with bladder cancer in Ahvaz.

## Materials and methods

### Participants and procedures

The present study was a cross-sectional study that examined the relationship between the oral health literacy and the DMFT index with the oral health – related quality of life in patients with bladder cancer at Golestan Hospital in Ahvaz as a public center of cancer in Southwest of Iran, 2023. The study population consisted of all patients with bladder cancer registered at the Cancer Registry Center of Khuzestan Province. The samples were randomly selected from the list of bladder cancer patients those were registered in Cancer Registry Center in Ahvaz Jundishapur University of Medical sciences and invited to Golestan Hospital for data collection through clinical evaluation, the Oral Health Literacy Adult Questionnaire (OHL-AQ), and the Oral Health Impact Profile-14 (OHIP-14PER) questionnaire. Also those patients who were willing to participate in the study and met the inclusion criteria were included in the study after providing informed consent. The inclusion criteria included were having bladder cancer and willingness to participate in the study. The exclusion criteria were unwillingness to continue the research or incomplete questionnaires. The sample size was determined based on the findings of previous studies and using the statistical software Med Calk with a power of 80% and a 5% error rate, 192 individuals were determined.

The data collection was after the study approved by the Ethics Committee in Ahvaz Jundishapur University of Medical Sciences (Ethics Code: IR.AJUMS.REC.1402.181).

### Measurement

#### Oral health literacy

The data on oral health literacy were collected through the Oral Health Literacy-Adults Questionnaire (OHL-AQ), which was specifically designed for the Iranian population and has been validated and reliable (15). This questionnaire consisted of 17 questions divided into 4 sections: 1. Comprehension, 2. Numeracy, 3. Listening Skills, and 4. Decision Making. To evaluate the responses, a correct answer was assigned a score of 1, while incorrect answers received a score of 0. Therefore, the total score for individuals ranged from 0 to 17. The higher the score, the higher the individual’s level of health literacy. The oral health literacy score is generally calculated between 0 and 17. Scores between 0 and 9 indicate insufficient oral health literacy, scores between 10 and 11 indicate borderline oral health literacy, and scores above 12 indicate sufficient oral health literacy (16). In addition, the questionnaire included questions about oral health behaviors, sources of oral health information, and demographic characteristics such as age, gender, education, and family members. The validity of the questionnaires was assessed using face and content validity, and the reliability of the questions was confirmed by experts using Cronbach’s alpha coefficient. The internal consistency, as measured by Cronbach’s alpha, was found to be 0.72, and the ICC was 0.84 (15).

#### Oral health-related quality of life

In order to assess the oral health-related quality of life, the Oral Health Impact Profile-14 (OHIP-14PER) questionnaire was used, which was first used by Slade in 1997 to examine seven aspects of oral health-related quality of life (17). This questionnaire consists of seven subgroups: functional limitation, physical pain, psychological discomfort, physical disability, psychological disability, social disability, and handicap, with each subgroup containing two questions. The responses were evaluated using the additive (ADD) method, in which the test options were scored as “never = 0, rarely = 1, sometimes = 2, often = 3, and always = 4.” In this method, the OHIP-14PER score ranged from 0 to 56, with lower scores indicating better quality of life for the individual. According to the study by Motallebnejad et al., this questionnaire has acceptable validity and reliability in the Iranian population (Cronbach’s alpha 0.095) (18).

### Statistical analysis

The data were analyzed using the statistical software SPSS 27 (IBM SPSS Statistics, Armonk, NY, United States). Qualitative variables were reported with frequency and percentage, while quantitative variables were reported with mean (standard deviation). The distribution data (Normal distribution) was examined using the Kolmogorov–Smirnov test, skewness, and kurtosis. To compare demographic and background variables within groups, the chi-square test and analysis of variance were used. Pearson correlation coefficient was used to examine the correlation between variables. A significance level of 0.05 was considered in all tests.

## Results

### Sample characteristics

All participants answered the questions in the survey and there were no missing data. There was a total of 194 participants, 104 individuals being male and the other half being female. Out of the participants, 145 individuals (74.7%) were married, while the remaining participants were single. 51individuals (26.3%) had at least one decayed-filled-extracted tooth. Other demographic and background variables are showed in Table 1.

**Table 1:** Demographic and background characteristics of the participants (*n* = 194).

Variable	Frequency	Percentage
Gender		
Male	104	53.6
Female	90	46.4
Age		
70–100	13	6.7
60–69	37	19.1
50–59	39	20.1
40–49	28	14.4
30–39	26	13.4
20–29	30	15.5
1–19	21	10.8
Education		
Illiterate	40	20.6
Elementary to diploma	138	70.2
University	16	8.2
Family Households		
1–5	154	79.4
6–10	40	20.6
Marital Status		
Single	49	25.3
Married	145	74.7
Job status		
Employed	63	32.5
Unemployed	114	58.7
Retired	17	8.8
Insurance status		
Yes	155	79.9
No	39	20.1
DMFT*		
Yes	51	26.3
No	143	73.7

*DMFT, decayed, missing, and filled teeth.

### Oral health literacy and oral health-related quality of life among participants

**Figure 1:**
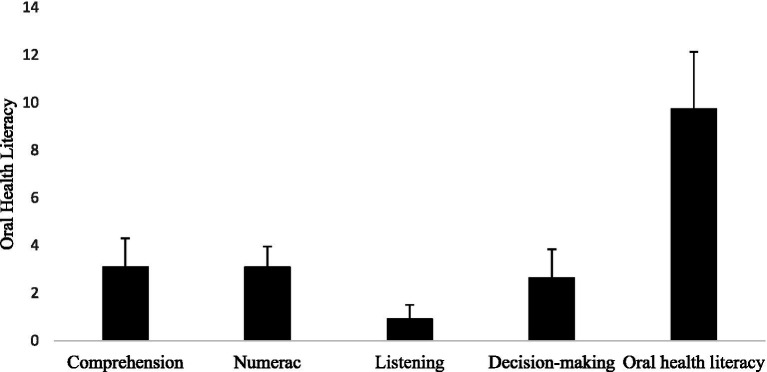
The mean score of oral health literacy and its dimensions among bladder cancer patients. The mean score of oral health literacy was 9.74 ± 2.39 which indicates insufficient oral health literacy levels. Scores between 0 and 9 indicate insufficient oral health literacy level.

The mean score of oral health literacy among bladder cancer patients was 9.74 ± 2.39 which indicates borderline oral health literacy levels (Figure 1).

In Table 2, the percentage of participants’ correct responses to each of the oral health literacy questions can be observed. Only about one-fourth of individuals answered questions 1, 3 (part 2), 9, and 13 correctly. In other words, a high percentage of individuals were unaware of the “connection between oral and heart diseases,” the “timing of the eruption of the first permanent tooth at around 6 years old,” the “duration of gas remaining in the mouth after tooth extraction,” and the meaning of the phrase “I exempt the dentist from unintended treatment outcomes on my tooth.” Approximately 53% of individuals were also unaware of the meaning of the phrase “I have a history of drug sensitivity or allergy.”

**Table 2:** Distribution of correct answers to the oral health literacy questionnaire.

Item	Correct answers percentage
1. The relationship between oral and dental diseases and Myocardial infarction” “	48 (24.7)
2.1. Preventing tooth decay by brushing with fluoride toothpaste.	77 (39.7)
2.2. Brushing with fluoride toothpaste twice a day.	155 (79.9)
2.3. Prevent tooth decay by reducing the consumption of sugary foods	158 (81.4)
3.1. The number of “permanent” teeth of each person is 32	122 (62.9)
3.2. Timing of the eruption of the first permanent tooth at around six years old	42 (21.6)
4. Time to take the next amoxicillin capsule following the diagnosis of dental infection and abscess	149 (76.8)
5. Continue taking the capsule even after the symptoms of the disease have disappeared	93 (47.9)
6. It is not possible to swallow sodium fluoride mouthwash	181 (93.3)
7. The permissible time for eating and drinking after using sodium fluoride mouthwash.	176 (90.7)
8. The duration of gas exiting the mouth after tooth extraction.	136 (70.1)
9. The time it takes to eat hot food after pulling a tooth.	39 (20.1)
10. The best course of action in case of slight bleeding from the gums after brushing or flossing	138 (71.1)
11. The best action in case of pain and swelling in the mouth	117 (60.3)
12. The most effective action to remove stains and discoloration from teeth	124 (63.9)
13. I understand the phrase of “I exempt the dentist from unintended treatment outcomes on my tooth.”	42 (21.69)
14. I understand the phrase of “I have a history of drug sensitivity or allergy”	93 (47.9)

According to the results of Table 3, there is a significant difference in the mean score of the oral health-related quality of life and its dimensions including functional limitation, physical pain, Psychological discomfort, physical disability, Psychological disability and Handicap between people with at least one DMFT and people without DMFT. Only in the dimension of social disability, people with at least one DMFT had a significantly lower oral health-related quality of life (6.09 ± 2.70) compared to people without DMFT (5.12 ± 2.62) (*p* = 0.025). Furthermore, there was no significant difference in the mean scores of oral health literacy domains, including numeracy, listening skills, and decision-making, between individuals with at least one DMFT and individuals without DMFT. In oral health literacy scores, Individuals without any DMFT had a significantly higher mean (9.98 ± 2.31) compared to individuals with DMFT (9.07 ± 2.50). Furthermore, the findings of the current study demonstrated that oral health literacy in the domain of comprehension was better in individuals without DMFT compared to those with such dental issues (Table 4).

**Table 3:** Oral health-related quality of life and its dimensions based on DMFT.

Variable	With DMFT^£^	Without DMFT	T^#^	*p* value
Functional limitation	5.49 ± 2.92	4.77 ± 2.50	−1.55	0.124
Physical pain	5.76 ± 2.89	5.17 ± 2.53	−1.289	0.201
Psychological discomfort	5.66 ± 2.89	4.97 ± 2.90	−1.453	0.148
Physical disability	5.58 ± 2.53	5.11 ± 2.55	−1.127	0.261
Psychological disability	6.37 ± 2.81	5.91 ± 2.63	−1.043	0.298
Social disability	6.09 ± 2.70	5.12 ± 2.62	−2.254	0.025*
Handicap	6.50 ± 2.86	5.67 ± 2.81	−1.820	0.070
OHRQOL^€^	41.49 ± 18.11	36.76 ± 16.44	−1.639	0.105

#Independent sample *t* test; £ DMFT, decayed, missing, and filled teeth; € OHRQOL, oral health-related quality of life; * *p* value < 0.05.

**Table 4:** Oral health literacy and its dimensions based on DMFT.

Variable	With DMFT^£^	Without DMFT	T^#^	*p* value
Comprehension	2.70 ± 1.33	3.24 ± 1.14	2.76	0.006*
Numeracy	2.94 ± 1.02	3.13 ± 0.82	1.33	0.184
Listening skills	0.96 ± 0.63	0.89 ± 0.60	−0.661	0.510
Decision making	2.47 ± 1.20	2.71 ± 1.19	1.24	0.216
Oral health literacy	9.07 ± 2.50	9.98 ± 2.31	2.35	0.020*

#Independent sample *t* test; £ DMFT, decayed, missing, and filled teeth; * *p* value < 0.05.

### Relationship between oral health literacy and oral health related quality of life among participants

The correlation matrix of the Pearson correlation coefficient between the dimensions of the Oral Health Impact Profile-14 Persian version (OHIP-14PER) and the Oral Health Literacy Assessment Questionnaire (OHL-AQ) is presented in Table 4.

The correlation analysis between the dimensions of the two questionnaires also showed that there is a significant negative correlation between OHRQOL and the dimensions of the oral health literacy questionnaire, except for the Listening Skills (*p* = 0.085). Also, the results of the correlation coefficient showed that the oral health literacy (*r* = −0.68) have correlation with the oral health-related quality of life (*r* = −0.68), and all dimensions of OHRQOL including functional limitations (*r* = −0.57), physical pain (*r* = −0.61), Psychological discomfort (*r* = −0.65), Psychological disability (*r* = −0.64), social disability (*r* = −0.60) and Handicap (*r* = −0.63) except physical disability (*r* = −0.57, *p* = 0.387) (Table 5).

**Table 5:** Pearson correlation coefficient matrix of oral health related-quality of life to oral health literacy in bladder cancer patients.

	Functional limitation	Physical pain	Psychological discomfort	Physical disability	Psychological disability	Social disability	Handicap	OHRQOL	Comprehension	Numeracy	Listening skill	Decision making	OHL
Functional limitation													
Physical pain	*r* = 0.67** p = 0.001												
Psychological discomfort	*r* = 0.70** p = 0.001	*r* = 0.71** p = 0.001											
Physical disability	*r* = −0.70** p = 0.001	*r* = 0.70** p = 0.001	*r* = 0.74** p = 0.001										
Psychological disability	*r* = −0.72** p = 0.001	*r* = 0.76** p = 0.001	*r* = 0.68** p = 0.001	*r* = 0.72** p = 0.001									
Social disability	*r* = −0.67** p = 0.001	*r* = 0.70** p = 0.001	*r* = 0.70** p = 0.001	*r* = 0.67** p = 0.001	*r* = 0.62** p = 0.001								
Handicap	*r* = −0.70** p = 0.001	*r* = 0.73** p = 0.001	*r* = 0.71** p = 0.001	*r* = 0.71** p = 0.001	*r* = 0.75** p = 0.001	*r* = 0.66** p = 0.001							
OHRQOL	*r* = 0.79** p = 0.001	*r* = 0.82** p = 0.001	*r* = 0.82** p = 0.001	*r* = 0.822** p = 0.001	*r* = 0.82** p = 0.001	*r* = 0.78** p = 0.001	*r* = 0.84** p = 0.001						
Comprehension	*r* = −0.41** p = 0.001	*r* = −0.40** p = 0.001	*r* = −0.49** p = 0.001	*r* = −0.30** p = 0.001	*r* = −0.38** p = 0.001	*r* = −0.47** p = 0.001	*r* = −0.47** p = 0.001	*r* = −0.47** p = 0.001					
Numeracy	*r* = −0.36** p = 0.001	*r* = −0.35** p = 0.001	*r* = −0.39** p = 0.001	*r* = −0.36** p = 0.001	*r* = −0.37** p = 0.001	*r* = −0.35** p = 0.001	*r* = −0.36** p = 0.001	*r* = −0.39** p = 0.001	*r* = 0.09 *p* = 0.170				
Listening skill	*r* = −0.16* p = 0.020	*r* = −0.135 *p* = 0.061	*r* = −0.08 *p* = 0.229	*r* = −0.11 *p* = 0.097	*r* = −0.18* p = -0.011	*r* = −0.12 *p* = 0.079	*r* = −0.10 *p* = 0.167	*r* = −0.12 *p* = 0.085	*r* = −0.05 *p* = 0.456	*r* = −0.008 *p* = 0.915			
Decision making	*r* = −0.41** p = 0.001	*r* = −0.46** p = 0.001	*r* = −0.44** p = 0.001	*r* = −0.48** p = 0.001	*r* = −0.51** p = 0.001	*r* = −0.39* p = 0.001	*r* = −0.44** p = 0.001	*r* = −0.52 *p* = 0.001**	*r* = 0.24** p = 0.001	*r* = 0.23** p = 0.001	*r* = 0.30** p = 0.001		
OHL	*r* = −0.57** p = 0.001	*r* = −0.61** p = 0.001	*r* = −0.65** p = 0.001	*r* = −0.57 *p* = 0.387	*r* = −0.64** p = 0.001	*r* = −0.60** p = 0.001	*r* = −0.63** p = 0.001	*r* = −0.68** p = 0.001	*r* = 0.63** p = 0.001	*r* = 0.51** p = 0.001	*r* = 0.36** p = 0.001	*r* = 0.77** p = 0.001	

OHRQOL, oral health-related quality of life; OHL, oral health literacy.

## Discussion

The results of the present study show that oral health literacy is borderline oral health literacy in patients with bladder cancer. Although a significant relationship was observed between oral health literacy and the DMFT index, no significant relationship was found between oral health-related quality of life and the DMFT index in patients with bladder cancer. Furthermore, a significant correlation was observed between oral health literacy and oral health-related quality of life and its dimensions (except for the functional disability dimension). In the following, the above findings were discussed in other studies and the possible reasons for observing these findings.

In the present study, it was demonstrated that oral health literacy was borderline among bladder cancer patients. This finding was consistent with the study by Batista et al. (19) that showed that show Approximately 71.5% of participant presented low OHL; but contradicted the study by Malek Mohammadi et al. (16) that showed The mean oral health literacy score was 12.07 (out of 17), and 62.5% of the participants had an adequate oral health literacy level. These different results seem to be due to different target groups, as in our study and Batista’s study (19), the participants were bladder cancer patients or older adult individuals, while in Malek Mohammadi et al.’s study (16), adult patients in dental clinics were assessed, and it is expected that their oral health literacy would be higher than patients and older adults in the community. In the present study, the mean oral health literacy score was 9.74 ± 2.39. In the study by Saied Moallemi et al. (20) in Isfahan, the mean oral health literacy score was 11.4 ± 3.4, and in the study by Naghavi et al., (21) the mean oral health literacy score was 10.5 ± 3.00 in Tehran. The reason for this may be lower education levels, differences in socio-economic class, and the presence of specific diseases. Overall, based on the present results, it appears that more efforts and interventions are needed to improve oral and dental health and increase oral health literacy in bladder cancer patients.

It would be important to relate job status to the values found, given that a large part of the population is unemployed (58.7%).

Based on the findings of the present study, oral health literacy is associated with all dimension’s oral health-related quality of life except physical disability. According to the findings of the current study, by Kimon Davaris et al., a strong correlation was found between oral health literacy of parents and their children’s oral health-related quality of life (13). In another study by Kimon Davaris et al., which was conducted only on low-income women, a weak and inverse relationship was also found between oral health literacy and oral health-related quality of life (22). It seems that higher health literacy can improve oral and dental health, as well as the oral health-related quality of life, in patients with bladder cancer, through increasing awareness, access to healthcare services, and the number of visits to the dentist.

Furthermore, the results of the present study showed a significant difference between the oral health literacy and DMFT index. These findings are consistent with the findings of Shayesteh et al., (*r* = −0.140, *p*-value = 0.047) (23) and are inconsistent with the findings of Yazdani et al., (*p* < −0.002) (24). Additionally, in the study by Amirchaghmaghi et al., (25), a significant negative and weak correlation was observed between the level of oral health literacy and the DMFT index (R = −0.127). This study demonstrated that individuals with inadequate oral health literacy had more decayed teeth and fewer filled teeth. The problem for patients with low oral health literacy in comprehension of guidelines and preventive recommendations may lead to less adherence to preventive recommendations. As a result, dental diseases, including decayed teeth, are more prevalent in these patients. It can be recommended to teach health literacy skills with the priority of reading comprehension in order to reduce the DMFT index.

No significant difference was observed between the oral health-related quality of life and DMFT index. However, only in the dimension of social disability, the DMFT index was associated with oral health-related quality of life. Similar to the results of another study among adolescents, no significant relationship was found between the DMFT index and the oral health-related quality of life (26). In a study in Sweden, no difference was found in oral health-related quality of life between young adults at high risk (DMFT>8) and low risk (DMFT = 0) of caries (27). On the other hand, Japanese university students with higher DMFT index had lower oral health-related quality of life (28). Furthermore, in a study by Drachev et al., it was shown that an increase in the DMFT index was significantly associated with lower oral health-related quality of life (OR = 1.05, 95% CI: 1.01–1.09) (29). The lack of a significant relationship between DMFT scores and oral health-related quality of life among bladder cancer patients may be due to the overall burden of bladder cancer reducing the impact of oral health issues on quality of life (30).

Finally, one notable finding is that 80% of the individuals were insured; however, dental services are often not covered by most insurance plans in Iran (30). Consequently, the out-of-pocket expenses for such services are significantly high, leading to a considerable unequally in this area. Introducing effective programs to facilitate access to dental services can be a key strategy in promoting oral health.

Based on our knowledge, the present study is the first study that examine oral health literacy and the oral health-related quality of life in bladder cancer patients. Some limitations should also be addressed. Firstly, we chose OHL-AQ for measuring the oral health literacy but other questionnaires are available and could have conducted to different results. Secondly, this selection bias is essentially unavoidable when using research registries but should be considered when interpreting results. Finally, there are potential factors that can influence the relationship between oral health literacy and the oral health-related quality of life, such as education and socio-economic status and other sources of bias, which were not considered in the study criteria and may have affected the results (31).

## Conclusion

According to the increasing prevalence of bladder cancer and the high rates of oral and dental diseases in bladder cancer patients, necessary planning should be carried out at a macro level in the field of prevention, care, and maintenance of oral and dental health. Overall, the results of this study indicate that the level of oral health literacy was not adequate in bladder cancer patients. Therefore, appropriate educational and psychological interventions and strategies should be implemented to improve oral health literacy and the oral health-related quality of life in bladder cancer patients. These findings emphasize the importance of considering oral health-related quality of life when developing intervention programs. Since all dimensions of oral health literacy affect oral health-related quality of life in bladder cancer patients, adopting oral hygiene behaviors and increasing oral health literacy can be the best way to improve oral health-related quality of life among bladder cancer patients.

## Data availability statement

The raw data supporting the conclusions of this article will be made available by the authors, without undue reservation.

## Ethics statement

The studies involving humans were approved by Iran Ahvaz Jundishapur University of Medical Sciences.REC.1402.181. The studies were conducted in accordance with the local legislation and institutional requirements. Written informed consent for participation in this study was provided by the participants' legal guardians/next of kin.

## Author contributions

FB: Data curation, Supervision, Writing – original draft. AA: Investigation, Project administration, Writing – original draft. MS Data Collection, Methodology. MC: Conceptualization, Formal analysis, Methodology, Supervision, Writing – review & editing.

## References

[1] Ferlay J Shin H Bray F Forman D Mathers C Parkin D. QOL comparison of PG and TG 415. Int J Cancer. (2010) 127:2893–917. doi: 10.1002/ijc.25516, PMID: 21351269

[2] Kalan Farmanfarma K Mahdavifar N Salehiniya H. Bladder cancer in Iran: an epidemiological review. Res Rep Urol. (2020) 5:91–103. doi: 10.2147/RRU.S232417 PMC7062394 32185152

[3] Nowroozi MR Amini E Farkhani EM Nowroozi A Ayati M Momeni SA. National and subnational incidence trend of bladder Cancer in Iran: report of a 12-year period. Int J Cancer Manag. (2023) 16:e127005. doi: 10.5812/ijcm-127005

[4] Lund Håheim L Thelle DS Rønningen KS Olsen I Schwarze PE. Low level of antibodies to the oral bacterium *Tannerella forsythia* predicts bladder cancers and *Treponema denticola* predicts colon and bladder cancers: a prospective cohort study. PLoS One. (2022) 17:e0272148. doi: 10.1371/journal.pone.0272148, PMID: 35994451 PMC9394794

[5] Oh H Lee DH Giovannucci EL Keum N. Gastric and duodenal ulcers, periodontal disease, and risk of bladder cancer in the health professionals follow-up study. Cancer Causes Control. (2020) 31:383–91. doi: 10.1007/s10552-020-01274-4, PMID: 32060837

[6] Michaud D Kelsey K Papathanasiou E Genco C Giovannucci E. Periodontal disease and risk of all cancers among male never smokers: an updated analysis of the health professionals follow-up study. Ann Oncol. (2016) 27:941–7. doi: 10.1093/annonc/mdw028, PMID: 26811350 PMC4843185

[7] Raber-Durlacher J Weijl N Abu Saris M De Koning B Zwinderman A Osanto S. Oral mucositis in patients treated with chemotherapy for solid tumors: a retrospective analysis of 150 cases. Support Care Cancer. (2000) 8:366–71. doi: 10.1007/s005200050004, PMID: 10975685

[8] Riantiningtyas RR Carrouel F Bruyas A Bredie WLP Kwiecien C Giboreau A. Oral somatosensory alterations in head and neck Cancer patients-an overview of the evidence and causes. Cancers (Basel). (2023) 15:718. doi: 10.3390/cancers15030718, PMID: 36765675 PMC9913236

[9] Saito H Watanabe Y Sato K Ikawa H Yoshida Y Katakura A. Effects of professional oral health care on reducing the risk of chemotherapy-induced oral mucositis. Support Care Cancer. (2014) 22:2935–40. doi: 10.1007/s00520-014-2282-4, PMID: 24854326 PMC4183888

[10] Petersen PE Kwan S. The 7th WHO global conference on health promotion-towards integration of oral health (Nairobi, Kenya 2009). Community Dent Health. (2010) 27:129–36. doi: 10.1922/CDH_2643Petersen08

[11] World Health Organization. Health literacy development for the prevention and control of noncommunicable diseases: Volume 4: Case studies from WHO national health literacy demonstration projects. (2022) 1:1–90.

[12] Bress LE. Improving Oral health literacy–the new standard in dental hygiene practice. J Dent Hyg. (2013) 87:322–9. 24357560

[13] Divaris K Lee JY Baker AD Vann WF Jr. Caregivers' oral health literacy and their young children's oral health-related quality-of-life. Acta Odontol Scand. (2012) 70:390–7. doi: 10.1177/0022034513484335 22150574 PMC3305855

[14] United States. Public Health Service. Office of the Surgeon General, National Institute of Dental and Craniofacial Research (US). Oral health in America: a report of the surgeon general: US public health service USA: Department of Health and Human Services (2000).

[15] Naghibi Sistani MM Montazeri A Yazdani R Murtomaa H. New oral health literacy instrument for public health: development and pilot testing. J Investig Clin Dent. (2014) 5:313–21. doi: 10.1111/jicd.12042, PMID: 23559571

[16] Mohammadi TM Malekmohammadi M Hajizamani HR Mahani SA. Oral health literacy and its determinants among adults in Southeast Iran. Eur J Dent. (2018) 12:439–42. doi: 10.4103/ejd.ejd_429_17 30147413 PMC6089060

[17] Slade GD. Derivation and validation of a short-form oral health impact profile. Community Dent Oral Epidemiol. (1997) 25:284–90. doi: 10.1111/j.1600-0528.1997.tb00941.x, PMID: 9332805

[18] Motallebnejad M Hadian H Mehdizadeh S Hajiahmadi M. Validity and reliability of the Persian version of the oral health impact profile (OHIP)-14. Caspian J Intern Med. (2011) 2:314–20. PMID: 24551438 PMC3895829

[19] Batista MJ Lawrence HP Sousa MLR. Oral health literacy and oral health outcomes in an adult population in Brazil. BMC Public Health. (2018) 18:1–9. doi: 10.1186/s12889-017-4443-0 PMC5530456 28747157

[20] Saied MZ Haghighi M. Assessing oral health literacy among the residents of Isfahan in 2014–2015. J Isfahan Dent Sch. (2016) 12:268–79.

[21] Sistani MMN Virtanen J Yazdani R Murtomaa H. Association of oral health behavior and the use of dental services with oral health literacy among adults in Tehran, Iran. Eur J Dent. (2017) 11:162–7. doi: 10.4103/ejd.ejd_332_16 28729786 PMC5502558

[22] Divaris K Lee JY Baker AD Vann WF. The relationship of oral health literacy with oral health-related quality of life in a multi-racial sample of low-income female caregivers. Health Qual Life Outcomes. (2011) 9:1–9. doi: 10.1186/1477-7525-9-108 22132898 PMC3248838

[23] Shayesteh M Shekarchizadeh H Rashidi MF. Investigation of Oral health literacy utilizing oral health literacy-adult questionnaire and its relationship with clinical indicators of oral health, as well as oral health behaviors, among dental patients. J Mash Dent Sch. (2022) 46:394–409. doi: 10.22038/jmds.2022.59780.2081

[24] Yazdani R Esfahani EN Kharazifard MJ. Relationship of oral health literacy with dental caries and oral health behavior of children and their parents. J Dent. (2018) 15:275–82. PMID: 30833973 PMC6397737

[25] Amirchaghmaghi M Movahhed T Mozaffari PM Torkaman F Ghazi A. Health literacy and its determinants in adult patients referred to dental clinics: a cross sectional study in Mashhad, Iran. Shiraz E Med J. (2019) 20:e86582. doi: 10.5812/semj.86582

[26] Biazevic MGH Rissotto RR Michel-Crosato E Mendes LA Mendes MOA. Relationship between oral health and its impact on quality of life among adolescents. Braz Oral Res. (2008) 22:36–42. doi: 10.1590/S1806-83242008000100007, PMID: 18425243

[27] Oscarson N Källestål C Lindholm L. A pilot study of the use of oral health-related quality of life measures as an outcome for analysing the impact of caries disease among Swedish 19-year-olds. Caries Res. (2007) 41:85–92. doi: 10.1159/000098040, PMID: 17284908

[28] Yamane-Takeuchi M Ekuni D Mizutani S Kataoka K Taniguchi-Tabata A Azuma T. Associations among oral health-related quality of life, subjective symptoms, clinical status, and self-rated oral health in Japanese university students: a cross-sectional study. BMC Oral Health. (2016) 16:1–8. doi: 10.1186/s12903-016-0322-9 27903265 PMC5129632

[29] Drachev SN Brenn T Trovik TA. Oral health-related quality of life in young adults: a survey of Russian undergraduate students. Int J Environ Res Public Health. (2018) 15:719–32. doi: 10.3390/ijerph15040719, PMID: 29641464 PMC5923761

[30] Saffarpour M Alaei Alamouti N. Assessment of the status of dental services in Iran (2018). Alborz Univ Med J. (2022) 11:241–55. doi: 10.29252/aums.11.2.241

[31] Saki F Cheraghi M Mohamadian H Ghorbanyjavadpour F. Oral health-related quality of life among narcotic and stimulant users referred to maintenance methadone therapy centers in Ahvaz city: Iran. Front Public Health. (2022) 10:5–19. doi: 10.3389/fpubh.2022.850550 PMC9163318 35669740

